# Renal ectopic fat deposition and hemodynamics in type 2 diabetes mellitus assessment with magnetic resonance imaging

**DOI:** 10.1186/s13244-025-01971-1

**Published:** 2025-04-27

**Authors:** Jian Liu, Hengzhi Chen, Chong Tian, Liwei Fu, Lisha Nie, Rongpin Wang, Xianchun Zeng

**Affiliations:** 1 Key Laboratory of Intelligent Medical Image Analysis and Precise Diagnosis of Guizhou Province, State Key Laboratory of Public Big Data, College of Computer Science and Technology, Guizhou University, No. 2870, Huaxi Avenue South, Guiyang, 550025 Guizhou China; 2 Department of Radiology, International Exemplary Cooperation Base of Precision Imaging for Diagnosis and Treatment, Guizhou Provincial People’s Hospital, No. 83, Zhongshan Dong Road, Guiyang, 550002 Guizhou China; 3 Department of Graduate School, Zunyi Medical University, Guizhou, Zunyi China; 4 GE HealthCare MR Research, Beijing, China

**Keywords:** Arterial spin labeling, IDEAL-IQ, Type 2 diabetes mellitus, Diabetic kidney disease

## Abstract

**Objectives:**

To assess renal perfusion and ectopic fat deposition in patients with type 2 diabetes mellitus (T2DM), and to evaluate the effects of ectopic fat deposition on renal hemodynamics.

**Methods:**

All participants underwent quantitative magnetic resonance imaging (MRI) to measure the cortical and medullary renal blood flow (RBF) and proton density fat fraction (PDFF). Patients with T2DM were classified into three groups according to the estimated glomerular filtration rate (mL/min/1.73 m^2^). One-way analysis of variance was used to assess differences among groups. Pearson’s correlation coefficient was used to analyze correlations. Additionally, a receiver operating characteristic (ROC) curve was constructed to assess diagnostic performance.

**Results:**

Renal PDFF values of the renal cortex and medulla, as well as perirenal fat thickness, were significantly different among the four groups: healthy control < T2DM < diabetic kidney disease (DKD) I–II < DKD III–IV. Additionally, significant differences in cortical and medullary RBF values were observed among the four groups: healthy control > T2DM > DKD I–II > DKD III–IV. A significant negative correlation was observed between renal PDFF and RBF values. Medullary RBF values demonstrated the best performance in discriminating T2DM from DKD with the largest area under the ROC curve (AUC) of 0.971. The cortical PDFF achieved the largest AUC (0.961) for distinguishing DKD I-II from DKD III–IV.

**Conclusions:**

Quantitative MRI effectively evaluates renal perfusion and ectopic fat deposition in T2DM patients, aiding in assessing kidney function and disease progression. Additionally, renal ectopic fat deposition may be an important risk factor for renal hemodynamic injury.

**Critical relevance statement:**

Quantitative MRI could serve as a radiation-free imaging modality for assessing renal perfusion and ectopic fat deposition, which may be an important risk factor for DKD progression.

**Key Points:**

- Quantitative MRI can be used to assess kidney function and monitor disease progression in patients with T2DM.
- In patients with T2DM, decreased renal perfusion, increased renal ectopic fat deposition, and kidney damage were significantly correlated.
- Renal ectopic fat deposition may be an important risk factor for renal hemodynamic injury.

**Graphical Abstract:**

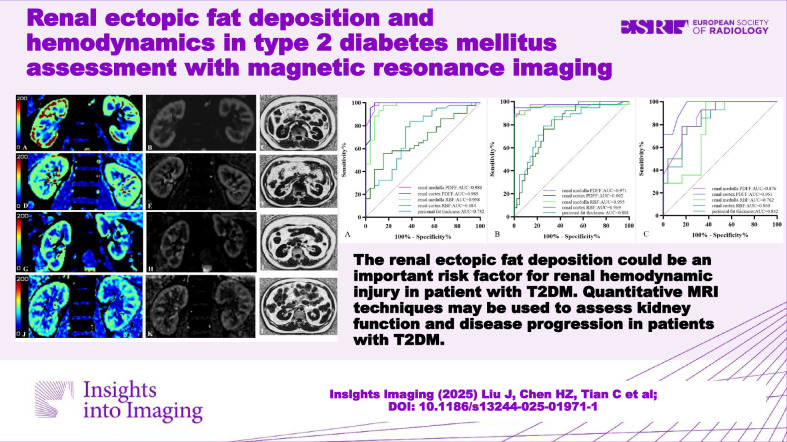

## Introduction

In recent decades, the global incidence of diabetes mellitus (DM) has rapidly increased, presenting a significant public health challenge associated with high costs and poor outcomes [1]. Diabetic kidney disease (DKD) is a common and complex microvascular complication of DM that affects approximately 20–40% of patients [2]. DKD is the primary cause of end-stage kidney disease and overall mortality in patients with DM, making it crucial to evaluate and understand its pathophysiology [2]. The impairment of the autoregulatory mechanism in the kidney contributes to DKD and is associated with reduced renal perfusion [3, 4]. Even in the early stages of DKD, hemodynamic changes have been observed, emphasizing the importance of precisely evaluating renal blood flow (RBF) reduction in patients with DM and understanding the factors that influence it [5].

Recent studies have identified fatty kidney disease (FKD) as a risk factor for reduced RBF in DKD [6]. FKD refers to the widespread accumulation of adipose tissue within the kidneys, including deposits in the renal hilum, sinus, and parenchyma [7, 8]. In patients with type 2 DM (T2DM), FKD plays a pivotal role in impairing renal perfusion [8–10]. Fat deposition surrounding the renal hilum and sinus directly compresses arterial blood flow, leading to decreased arterial inflow [7]. Aberrant fat deposition in the pararenal spaces and perirenal tissues further compresses the renal parenchyma and vessels, resulting in elevated renal interstitial hydrostatic pressure and a consequent reduction in tubular blood flow [11, 12]. In addition, patients with T2DM exhibit abnormal lipid droplet accumulation in the renal glomeruli, interstitium, and tubules, which correlates with impaired renal morphology and function [13, 14]. Fat deposition in the renal parenchyma contributes to glomerular impairment through lipotoxic effects and mechanical compression, while the adipokines produced exert proinflammatory and vasoconstrictive effects [15]. Collectively, these factors precipitate podocyte dysfunction and glomerulosclerosis, thereby causing hemodynamic changes. Renal biopsies have confirmed ectopic fat deposition in the kidneys of patients with T2DM [16, 17], underscoring the urgent need to investigate the relationship between renal perfusion and ectopic fat deposition, as the underlying mechanisms remain unclear.

Renal imaging has been suggested as a potential tool for evaluating the associations between perirenal and renal sinus fat and renal blood perfusion [18]; however, its precise role in assessing the correlation between renal parenchymal fat deposition and renal perfusion remains unclear. There is growing interest in the exploration of noninvasive auxiliary or alternative techniques to assess renal function. Recent advancements in renal magnetic resonance imaging (MRI) have facilitated the assessment of renal ectopic fat deposition and RBF during the progression of DKD. Arterial spin labeling (ASL) imaging, which employs intrinsic water molecules as tracers, has been extensively used in cerebral imaging [3]. Recent studies have reported the usefulness and accuracy of ASL for quantifying RBF in humans [19, 20], and ASL enables direct assessment of renal perfusion in patients with DKD [19, 21, 22]. In addition, we previously validated the reproducibility of renal parenchymal fat measurement using the iterative decomposition of water and fat with echo asymmetry and a least-squares estimation-iron quantification (IDEAL-IQ) sequence [23].

Therefore, we used quantitative MRI to assess the progression of hemodynamic damage associated with kidney disease advancement in individuals with T2DM and examined the impact of renal ectopic fat deposition on RBF.

## Methods

### Participants

**Fig. 1:**
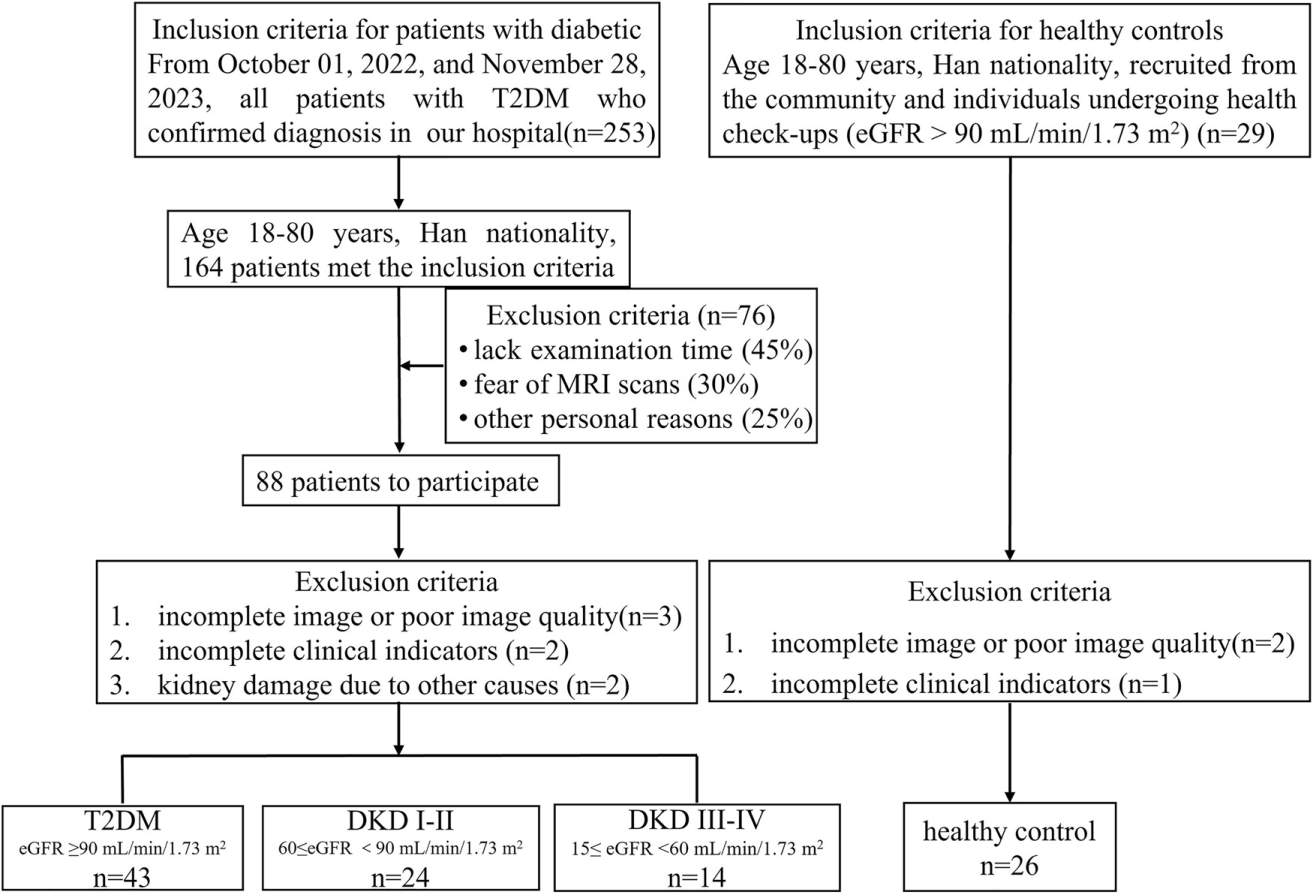
Main inclusion and exclusion criteria for the study participants. T2DM, type 2 diabetes mellitus; eGFR, estimated glomerular filtration rate; DKD, diabetic kidney disease

The prospective study was conducted in accordance with the Declaration of Helsinki (as revised in 2013) and was approved by the Institutional Review Board (approval number: KY 2022-02). Written informed consent was obtained from all participants prior to MRI evaluation. Participants were recruited from the endocrinology and nephrology outpatient clinics and inpatient wards of our hospital between October 1, 2022, and November 28, 2023. A continuous recruitment strategy was employed, in which all patients with a confirmed diagnosis of T2DM visiting these departments during the study period were screened for eligibility. During the recruitment period, 253 patients were screened, of whom 164 met the inclusion criteria and were invited to participate. Of the invited patients, 88 agreed to participate, resulting in a 54% participation rate. Baseline characteristics of participants and non-participants were compared, and no statistically significant differences were observed, indicating minimal selection bias. The final analysis included 107 participants, comprising 26 healthy controls (HCs) and 81 patients with T2DM. Patients with T2DM were confirmed according to the American Diabetes Association [24, 25] guidelines prior to study enrollment, and all were aged ≥ 18 years. The diagnosis of DKD was determined based on the treating physician’s assessment, which considered pathological biopsy findings or evaluations of estimated glomerular filtration rate (eGFR), urine albumin: creatinine ratio (UACR), and T2DM duration, among other factors [25]. Healthy volunteers were recruited from the community and from individuals undergoing routine health check-ups at our hospital. Control participants exhibited an eGFR > 90 mL/min/1.73 m^2^, normal urine analysis findings, and no history of hypertension, DM, or kidney disease. Exclusion criteria included contraindications to MRI (e.g., the presence of metallic or mechanical implants, claustrophobia, or electronic devices) and other conditions that could impair kidney function, such as ongoing immunosuppressive therapy, acute or chronic inflammatory diseases, or active autoimmune or malignant conditions. Detailed inclusion and exclusion criteria are summarized in Fig. 1.

### Clinical evaluation

Medical histories and physical examinations were conducted for all participants. The recorded parameters included age, gender, height, and weight. Urine and blood samples were collected from all patients to determine serum creatinine (Scr) and urinary albumin levels. The eGFR was calculated using the Modification of Diet in Renal Disease Study equation [26]. Participants with T2DM were stratified by eGFR according to the Kidney Disease: Improving Global Outcomes classification of CKD [25]. Patients were classified based on eGFR (mL/min/1.73 m^2^) into group I (eGFR > 90 mL/min/1.73 m^2^, T2DM), group II (90 mL/min/1.73 m^2^ > eGFR > 60 mL/min/1.73 m^2^, DKD stage I–II), or group III (60 mL/min/1.73 m^2^ > eGFR > 15 mL/min/1.73 m^2^, DKD stages III–IV).

### MRI protocols

The investigations were performed using a 3.0-T MRI scanner (Discovery MR 750W, GE Healthcare, Milwaukee, WI, USA) equipped with an eight-channel abdominal surface receiver coil. Participants were instructed to remain still throughout the scanning procedure. The MRI protocol included axial and coronal T2-weighted single-shot fast spin-echo sequences (T2WI) to detect significant kidney structural abnormalities. The renal proton-density fat fraction (PDFF) was acquired using a breath-hold IDEAL-IQ at the end of expiration. The imaging parameters were as follows: echo time (TE) = 2.5 ms, repetition time (TR) = 6.2 ms, matrix size = 160 × 160, slice thickness = 5 mm, field of view (FOV) = 420 × 336 mm, and acquisition time = 17 s. Perfusion-weighted images were acquired using a pulsed ASL sequence during a breath-hold at end expiration. The axial labeling plane was positioned perpendicular to the aorta, approximately 10 cm above the renal region. The labeling parameters included a labeling duration of 1.6 s and a post-labeling delay of 1.5 s. The imaging parameters for the ASL sequence were as follows: TR/TE = 7000/19 ms, FA = 90°, FOV = 380 mm × 380 mm, slices = 7 mm, and matrix = 96 × 96.

### Image analysis

The image quality was evaluated by the first author. The regions of interest (ROIs) were individually delineated by two experienced radiologists (C.T., 9 years of experience; H.Z.C., 3 years of experience) using the communication system (AW.4.6) software. Reviewers were unaware of their clinical information. T2WI was used as an anatomical reference to distinguish between the renal cortex and medulla. The six ROIs were carefully placed on the cortex and medulla of the renal hilum, with an area of approximately 10–25 mm^2^, avoiding the renal sinus, large blood vessels, and perirenal tissue. For each kidney, the cortical and medullary PDFF values were calculated as the mean values of the six ROIs. Because the biochemical parameters measured represented the overall function of both kidneys, the cortical and medullary PDFF values of the kidneys were averaged [27]. Simultaneously, the perirenal fat thickness was measured by determining the distance from the anterior margin of the quadratus lumborum muscle to the dorsal margin of the kidney on the central slice of the renal hilum. Quantitative RBF maps were generated after data acquisition, adhering to a formula established in earlier studies [28, 29]. Using this map, RBF was quantified and expressed in mL/100 g/min, using the following equation:

1 $$\Delta M \, = 	 \, 2\frac{{M}_{0}}{\lambda }f\frac{1}{{R}_{1app}}\alpha \exp (\frac{-\Delta t}{{T}_{1,blood}})\cdot \exp (-(t-\tau -\Delta t){R}_{1app})\cdot \\ 	 (1-\exp (-\tau {R}_{1app}))$$

2 $${{\rm{R}}}{{\rm{B}}}{{\rm{F}}}=\frac{{{\Delta }}M}{{M}_{0}}\frac{\lambda }{2\alpha }\frac{{R}_{1app}}{\exp (-\omega {R}_{1app})-\exp (-(\tau +\omega ){R}_{1app})}$$

3 $${R}_{1app}={R}_{1}+\frac{f}{\lambda }$$

where Δ*M* represents the differential signal between labeled and control images, *M*_o_ denotes the mean intensity of control images, *λ*(0.9 mL/g) refers to the blood-to-tissue water partition coefficient, *f* (mL/100 g/min) is RBF, *α* (0.75) indicates the efficiency of labeling, Δ*t* is the arrival time of labeled arterial blood, *T*_1, blood_ is the longitudinal relaxation time of arterial blood, *R*_1app_ is the longitudinal relaxation rate of arterial blood flow, *τ* (1.6 s) is the labeling pulse duration, and *R*_1_ is the longitudinal relaxation rate in the absence of blood flow.

**Fig. 2:**
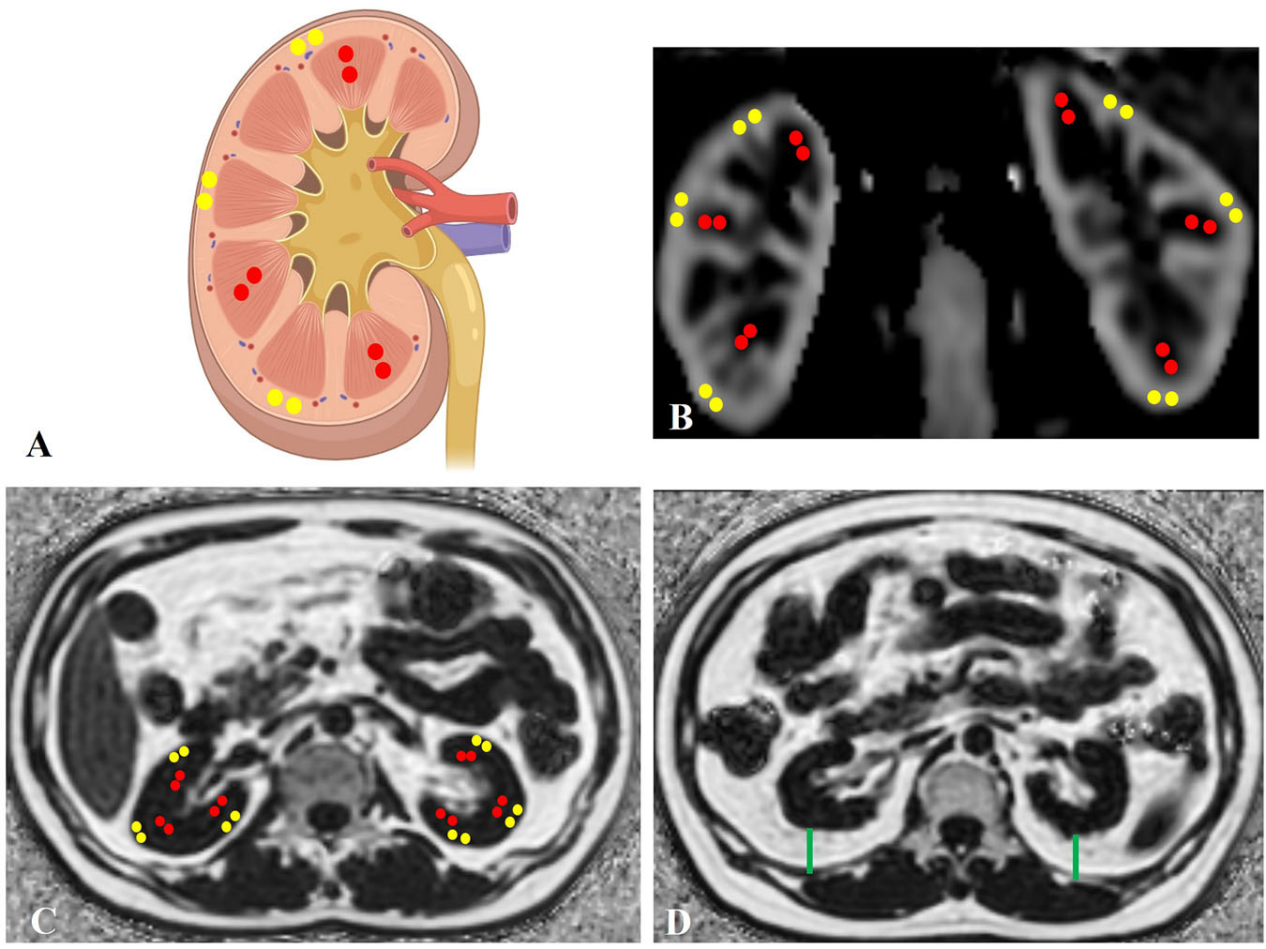
RBF and PDFF values of cortex and medulla and perirenal fat thickness were measured in the central slice of renal hilum. **A** Two ROIs were selected per kidney in the renal cortex (yellow circle) and medulla (red circle) upper, middle, and lower poles on the schematic map; **B** Six ROIs were manually placed per kidney cortex (yellow circle) and medulla (red circle) on the RBF map. **C** Six ROIs were selected per kidney in the renal cortex (yellow circle) and medulla (red circle) on the fat fraction map. **D** Perirenal fat thickness was calculated by the distance from the anterior margin of the quadratus lumborum muscle to the dorsal margin of the kidney

Because the renal medulla is particularly susceptible to ischemia and hypoxia, we evaluated both cortical and medullary RBF values. To avoid including the collecting system and vessels, six ROIs were manually positioned at the upper, middle, and lower poles of each renal cortex and medulla on the RBF maps. For analysis, two coronal planes near the renal hilum were selected. The cortical and medullary RBF values were calculated by averaging the pixel values across both kidneys. Typical placements of the ROIs are shown in Fig. 2.

### Statistical analysis

Statistical analysis was conducted using the SPSS software package version 26.0. Normally distributed continuous variables were presented as mean ± standard deviation. Non-normally distributed variables were depicted using medians and interquartile ranges, with comparisons performed using the Kruskal–Wallis *H*-test. Categorical variables were expressed as counts and compared using the chi-square test. The intraclass correlation coefficient (ICC) was calculated to assess interobserver agreement in RBF and PDFF measurements. Right, left, and cortical-medullary renal RBF and PDFF values were compared using paired *t*-tests. Differences in renal PDFF values and RBF values among the four groups were examined using one-way analysis of variance. Relationships between variables were assessed using Pearson’s correlation analysis. Diagnostic performance was evaluated by comparing areas under the receiver-operating-characteristic (ROC) curves. All analyses were two-tailed, and statistical significance was set at *p* < 0.05.

## Results

### Clinical and laboratory characteristics

Ten participants (seven with T2DM and three healthy volunteers) were excluded from the analysis due to poor imaging quality or incomplete clinical indicators. The final study population comprised 26 healthy volunteers and 81 patients with T2DM (63 males and 44 females). Baseline data of the participants are presented in Table 1, which includes typical characteristics, such as age, gender, body mass index (BMI), waist-to-hip ratio, UACR, eGFR, Scr, and microalbuminuria. The gender distribution and BMI among the four groups were not significantly different (gender, *p* = 0.423; BMI, *p* = 0.108).

**Table 1:** Characteristics of the participants

	HC	T2DM	DKDI–II	DKDIII–IV	*F*	p*
Gender (M/F)	14/12	25/18	16/8	8/6	6.000^*χ*2^	0.423
Age (years)	56.17 ± 8.95	52.53 ± 9.45	57.25 ± 8.51^a^	60.86 ± 9.03^a^	3.462*	0.020
BMI (kg/m^2^)	23.23 ± 2.76	24.61 ± 3.09	25.38 ± 3.26	26.25 ± 4.31	2.087*	0.108
WHR	0.95 ± 0.03	0.91 ± 0.06	0.94 ± 0.07	0.98 ± 0.06^a,b^	5.452*	0.002
Diabetes duration (years)	-	6.35 ± 5.69	9.84 ± 4.64^a^	11.07 ± 7.11^a^	5.076*	0.008
UACR	-	0.59 (0.15, 1.09)	2.97 (0.68, 11.71)^a^	69.95 (9.39, 391.96)^a,b^	27.789^①^	< 0.001
eGFR (mL/min/1.72 m^2^)	-	103.00 (96.00, 109.00)	86.00 (77.50, 100.50)^a^	46.50 (39.75, 52.75)^a,b^	50.326^①^	< 0.001
Scr (µmol/L)	-	62.00 (51.00, 74.00)	76.00 (66.75, 91.00)^a^	137.50 (123.75, 155.50)^a,b^	46.013^①^	< 0.001
Microalbuminuria	-	3.71 (1.45, 11.90)	24.05 (5.16, 137.59)^a^	300.71 (62.62, 2384.53)^a,b^	32.462^①^	< 0.001

Notes: Data are presented as the number, mean ± standard deviation, or median (first quartile, third quartile)

* LSD multiple pairwise comparison tests were used for statistical significance ANOVA. ^*χ*2^ Chi-square test

*HC* healthy control, *T2DM* type 2 diabetes mellitus, *DKD* diabetic kidney disease, *BMI* body mass index, *WHR* waist-hip ratio, *UACR* urine albumin-to-creatinine ratio, *eGFR* estimated glomerular filtration rate, *Scr* serum creatinine

^①^ Kruskal–Wallis *H*-tests. Means with different letters indicate significant differences between the groups

^a^ Significant difference from T2DM (*p*  < 0.05)

^b^ Significant difference from DKDI-II (*p*  < 0.05)

After analyzing the MR images, we observed excellent consistency between the RBF and PDFF values measurements by the two radiologists, with ICCs of 0.866 (95% confidence interval: 0.822–0.900) and 0.822 (95% confidence interval: 0.774–0.861), respectively. Table 2 presents the mean values ± standard deviation of renal PDFF and RBF values for each group. In all four groups, cortical RBF values were significantly higher than the medullary RBF values, whereas there was no significant difference between the cortical and medullary PDFF values (RBF: *t* = −35.985, *p* < 0.001; PDFF: *t* = −0.825, *p* = 0.411).

**Table 2:** Comparison of PDFF, RBF values in renal cortex and medulla, and perirenal fat thickness in four groups (x̅ ± s)

Group	PDFF (%)		RBF (mL/100 g/min)		Perirenal fat thickness (mm)
	Cortex	Medulla	Cortex	Medulla	
HC	0.88 ± 0.09	0.86 ± 0.10	182.97 ± 47.29	42.62 ± 4.11	6.39 ± 3.18
T2DM	1.22 ± 0.09^a^	1.18 ± 0.09^a^	158.61 ± 29.03^a^	35.71 ± 1.45^a^	10.33 ± 5.79^a^
DKDI-II	1.53 ± 0.13^a,b^	1.56 ± 0.14^a,b^	137.04 ± 14.57^a,b^	30.59 ± 2.97^a,b^	14.49 ± 6.31^a,b^
DKDIII-IV	1.74 ± 0.08^a,b,c^	1.75 ± 0.10^a,b,c^	116.23 ± 13.55^a,b,c^	27.79 ± 1.67^a,b,c^	23.65 ± 6.27^a,b,c^
*F*	300.233	288.135	17.287	124.587	31.848
p*	< 0.001	< 0.001	< 0.001	< 0.001	< 0.001

Notes: Data are presented as the number, mean ± standard deviation. Means with different letters indicate significant differences between the groups

* LSD multiple pairwise comparison tests were used for statistical significance ANOVA

*RBF* renal blood flow, *PDFF* proton-density fat fraction, *HC* Healthy control, *T2DM* type 2 diabetes mellitus, *DKD* diabetic kidney disease

^a^ Significant difference from HC (*p*  < 0.05)

^b^ Significant difference from T2DM (*p*  < 0.05)

^C^ Significant difference from DKDI-II (*p*  < 0.05)

### Renal perfusion and fat deposition

**Fig. 3:**
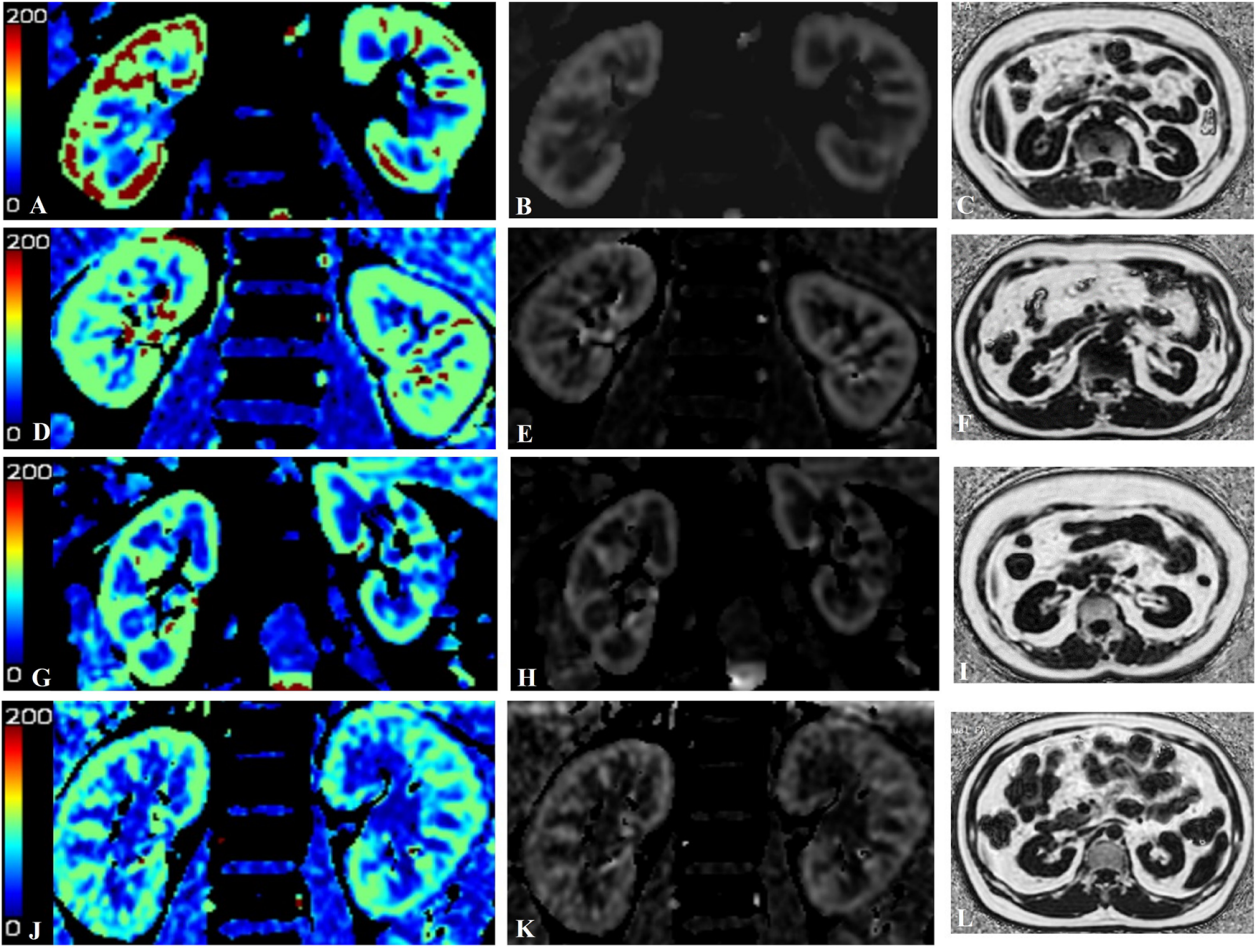
Comparison of RBF and PDFF values of cortex, medulla, and perirenal fat thickness. **A**–**C** A 62-year-old woman with healthy kidneys, where the mean renal RBF were 167.09 mL/100 g/min (Cortex) and 42.37 mL/100 g/min (Medulla), the renal parenchyma PDFF were 0.78% (Cortex) and 0.81% (Medulla) and the thickness of perirenal fat is 6.13 mm. **D**–**F** A 58-year-old woman with T2DM, where the mean renal RBF were 152.78 mL/100 g/min (Cortex) and 32.58 mL/100 g/min (Medulla), the renal parenchyma PDFF were 1.15% (Cortex) and 1.19% (Medulla) and the thickness of perirenal fat is 9.59 mm. **G**–**I** a 67-year-old man with stage 2 DKD, where the mean renal RBF were 139.63 mL/100 g/min (Cortex) and 28.56 mL/100 g/min (Medulla), the renal parenchyma PDFF were 1.58% (Cortex) and 1.51% (Medulla) and the thickness of perirenal fat is15.98 mm. **J**–**L** a 62-year-old man with stage 4 DKD, where the mean renal RBF were134.86 mL/100 g/min (Cortex) and 26.56 mL/100 g/min (Medulla), the renal parenchyma PDFF were 1.69% (Cortex) and1.68% (Medulla), and the thickness of perirenal fat is 22.35 mm. **A**, **D**, **G**, **I** = RBF color images; **B**, **E**, **H**, **K** = RBF maps; **C**, **F**, **I**, **L** = fat fraction maps. Higher renal cortical perfusion and lower PDFF values are shown in images (**A**–**C**)

The mean RBF values, mean renal PDFF values, and perirenal fat thickness measured in healthy volunteers and in patients with T2DM stratified by disease stages are shown in Fig. 3 and reported in Table 2. Furthermore, the renal cortical and medullary PDFF values, as well as the perirenal fat thickness, differed significantly among the four groups: healthy control < T2DM < DKD I–II < DKD III–IV. In addition, significant differences in the cortical and medullary RBF values were observed among the four groups: healthy control > T2DM > DKD I–II > DKD III–IV.

Table 3 shows the mean values ± standard deviation values for PDFF values, RBF, and perirenal fat thickness for the left and right kidneys. No significant differences were observed between the left and right kidneys in terms of PDFF values (cortex: *p* = 0.632; medulla: *p* = 0.641), RBF values (cortex: *p* = 0.065; medulla: *p* = 0.216), or perirenal fat thickness (*p* = 0.114).

**Table 3:** Comparison of PDFF and RBF values in the renal cortex and medulla, and perirenal fat thickness between the left and right kidneys in all participants (x̅ ± s)

	Left renal	Right renal	*t*	*p*^#^
Cortex PDFF (%)	1.26 ± 1.99	1.27 ± 2.18	−0.481	0.632
Medulla PDFF (%)	1.25 ± 1.99	1.26 ± 2.18	−0.468	0.641
Cortex RBF (mL/100 g/min)	152.94 ± 37.83	155.35 ± 38.13	−1.868	0.065
Medulla RBF (mL/100 g/min)	34.54 ± 9.49	34.87 ± 10.07	1.243	0.216
Perirenal fat thickness (mm)	12.38 ± 8.17	11.83 ± 7.29	1.592	0.114

Notes: Data are presented as the number, mean ± standard deviation

*RBF* renal blood flow, *PDFF* proton-density fat fraction

^#^ Paired t-test were used for comparison

### Correlation analysis

Table 4 presents the correlation between renal PDFF values, RBF values, and biochemical parameters. For patients with T2DM and DKD (combined), significant negative correlations were observed between the PDFF and RBF values (cortical PDFF with cortical RBF, *r* = −0.640; cortical PDFF with medullary RBF, *r* = −0.910; medullary PDFF with cortical RBF, *r*0 = −0.636; medullary PDFF with medullary RBF, *r* = −0.849). In addition, significant negative correlations were found between PDFF values and eGFR (cortex, *r* = −0.711; medulla, *r*  = −0.734). Furthermore, significant positive correlations were observed between PDFF values and perirenal fat thickness (cortex, *r* = 0.670; medulla, *r* = 0.644), Scr (cortex, *r* = 0.709; medulla, *r* = 0.714), and microalbuminuria (cortex, *r* = 0.481; medulla, *r* = 0.603). Moreover, significant positive correlations were observed between the RBF values and eGFR (cortex, *r* = 0.467; medulla, *r* = 0.685), as well as significant negative correlations between RBF values and perirenal fat thickness (cortex, *r* = −0.479; medulla, *r* = −0.595), Scr (cortex, *r* = −0.467; medulla, *r* = −0.671), and microalbuminuria (cortex, *r* = −0.343; medulla, *r* = −0.519).

**Table 4:** Correlation of renal cortical and medullary PDFF and RBF values

Group	PDFF (%)				RBF (mL/100 g/min)			
	Cortex		Medulla		Cortex		Medulla	
	*r*	*p*	*r*	*p*	*r*	*p*	*r*	*p*
Cortex PDFF	-	-	0.888	< 0.001	−0.640	< 0.001	−0.910	< 0.001
Medulla PDFF	0.888	< 0.001	-	-	−0.636	< 0.001	−0.849	< 0.001
Cortex RBF	−0.640	< 0.001	−0.636	< 0.001	-	-	0.704	< 0.001
Medulla RBF	−0.910	< 0.001	−0.849	< 0.001	0.704	< 0.001	-	-
Perirenal fat	0.670	< 0.001	0.644	< 0.001	−0.479	< 0.001	−0.595	< 0.001
eGFR (mL/min/1.72 m^2^)	−0.711	< 0.001	−0.734	< 0.001	0.467	< 0.001	0.685	< 0.001
Scr (µmol/L)	0.709	< 0.001	0.714	< 0.001	−0.467	< 0.001	−0.671	< 0.001
Microalbuminuria	0.481	< 0.001	0.603	< 0.001	−0.343	< 0.001	−0.519	< 0.001

*RBF* renal blood flow, *PDFF* proton-density fat fraction, *eGFR* estimated glomerular filtration rate, *Scr* serum creatinine

### Diagnostic performance of parameters

**Fig. 4:**
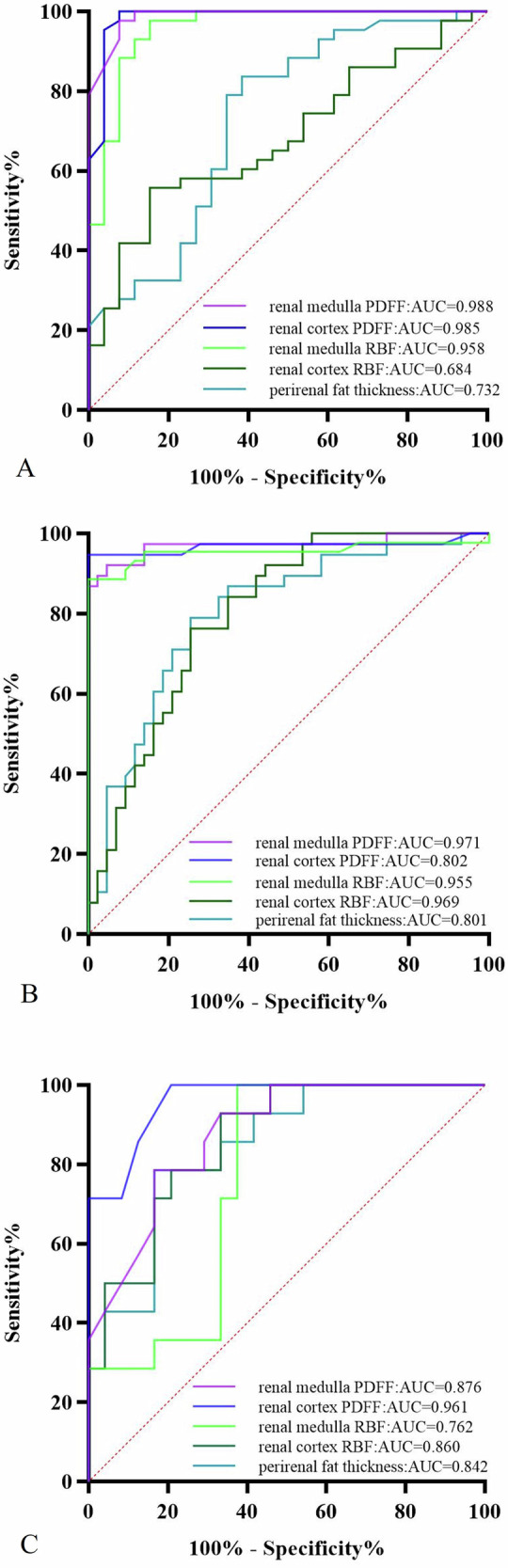
ROC curve for discriminating HC from T2DM (**A**), T2DM from DKD (**B**), and DKD stage I–II from DKD stage III–IV (**C**). ASL and IDEAL-IQ demonstrate strong diagnostic accuracy in assessing the severity of renal damage in patients with T2DM. The specific diagnostic capabilities are as follows: discriminating T2DM from DKD > discriminating HC from T2DM > discriminating DKD I–II from DKD III–IV. HC, healthy control; T2DM, type 2 diabetes mellitus; DKD, diabetic kidney disease

The ROC curves for cortical and medullary PDFF, RBF, and perirenal fat thickness in discriminating among the four groups are presented in Fig. 4, and the corresponding diagnostic values are shown in Table 5. Among the PDFF and RBF values of the renal cortex, medulla, and perirenal fat thickness, the medullary PDFF values exhibited the best performance in discriminating T2DM from the controls, the medullary RBF values demonstrated the highest diagnostic accuracy in differentiating T2DM from DKD, and the renal cortical PDFF value showed the best performance in discriminating DKD stage I–II from DKD stage III–IV.

**Table 5:** Diagnostic performance of the renal PDFF, RBF values, and perirenal fat thickness for the discrimination among the four groups

Parameters	Cut-off	AUC	*p*	Sensitivity /%	Specificity /%
RBF value of the renal cortex					
Discriminating HC from T2DM	160.7	0.684	0.011	55.81	84.62
Discriminating T2DM from DKD	140.5	0.802	< 0.001	76.32	74.42
Discriminating DKD I–II from DKD III–IV	136.8	0.860	< 0.001	92.86	62.50
RBF value of the renal medulla					
Discriminating HC from T2DM	39.18	0.958	< 0.001	97.67	84.62
Discriminating T2DM from DKD	33.64	0.971	< 0.001	92.11	95.35
Discriminating DKD I-II from DKD III–IV	29.97	0.762	0.008	100	62.5
PDFF value of the renal cortex					
Discriminating HC from T2DM	1.03	0.985	< 0.001	100	92.31
Discriminating T2DM from DKD	1.41	0.969	< 0.001	94.74	100
Discriminating DKD I-II from DKD III–IV	1.64	0.961	< 0.001	100	79.27
PDFF value of the renal medulla					
Discriminating HC from T2DM	1.05	0.988	< 0.001	97.68	92.33
Discriminating T2DM from DKD	1.38	0.955	< 0.001	88.64	100
Discriminating DKD I-II from DKD III–IV	1.69	0.876	< 0.001	78.57	83.33
Perirenal fat thickness		-			
Discriminating HC from T2DM	6.18	0.732	0.001	83.72	61.54
Discriminating T2DM from DKD	11.33	0.801	< 0.001	78.95	74.42
Discriminating DKD I-II from DKD III–IV	19.43	0.842	< 0.001	78.57	83.33

*HC* healthy control, *T2DM* type 2 diabetes mellitus, *DKD* diabetic kidney disease, *AUC* areas under curves, *RBF* renal blood flow, *PDFF* proton-density fat fraction

## Discussion

In this study, we investigated the relationship between renal ectopic fat deposition and hemodynamics impairment by applying ASL and IDEAL-IQ imaging. To our knowledge, this represents the first study to characterize the impact of intrarenal parenchymal fat accumulation on renal perfusion deficits in patients with T2DM. Our findings suggest that both renal perfusion parameters and ectopic fat deposition may serve as prognostic biomarkers for DKD, facilitating early diagnosis of renal dysfunction and the identification of progressive disease trajectories. ASL and IDEAL-IQ show great promise as components of multiparametric studies of renal structure and function, and they have the potential to inform drug development while providing clinically relevant information.

Impairment of renal perfusion combined with renal ectopic fat deposition has been identified as a marker of kidney damage in patients with T2DM [8, 30]. However, evaluating kidney microvasculature perfusion and renal parenchymal fat deposition remains challenging, as conventional methods are both invasive and time-consuming [19]. Consequently, these methods are not widely used in clinical practice. Recently, non-invasive renal quantitative MRI techniques have emerged as promising approaches for measuring physiological markers in the kidney. In our recent study, we established the reproducibility and reliability of IDEAL-IQ for measuring renal parenchymal fat deposition [23]. IDEAL-IQ accounts for T2* inhomogeneity and yields PDFF measurements that are not confounded by iron overload. Additionally, this technique requires shorter scan times compared to conventional [31]. Additionally, ASL has shown significant promise in the non-invasive measurement of regional perfusion in the kidneys [32]. This method is particularly appealing for RBF measurement because it utilizes magnetically labeled arterial blood water as a tracer, thereby eliminating the need for exogenous contrast agents [19]. The signal from ASL correlates directly with RBF values and enables the quantification of renal perfusion in physiological units (mL/100 g/min) [28]. Over the past few years, its reproducibility and its association with conventional kidney methods have validated ASL as a novel and reliable approach for evaluating renal perfusion in both animals and humans [33].

This study represents one of the first applications of renal ASL and IDEAL-IQ to quantify changes in kidney microvascular perfusion and renal parenchymal fat deposition in patients with T2DM. The renal tissue perfusion ASL results observed in healthy volunteers in the present study are consistent with those in previous reports [30, 34]. Previous studies reported renal cortical perfusion values ranged from 139 to 427 mL/100 g/min in the healthy cohorts [20, 35–37]. This variability can be partially attributed to differences in population characteristics and measurement techniques (such as field strength and acquisition methods). In addition, the renal cortex receives significantly higher blood perfusion than the medulla [38], a difference attributed to the rich vascular supply of the cortex compared to the medulla. However, hyperglycemia-induced endothelial dysfunction and subsequent microvascular rarefaction may decrease blood flow and increase oxygen consumption, leading to hypoxia. In renal dysfunction, hypoxia plays a critical role in disease progression, with the medulla being more vulnerable than the cortex. Therefore, it is essential to measure renal medullary perfusion separately. Similarly, cortical and medullary PDFF values should also be assessed independently. Nevertheless, due to limited image quality, previous studies have primarily focused on global renal parenchymal fat deposition [16, 17, 39]. In this study, PDFF values in the renal cortex and medulla were measured using T2WI as an anatomical reference, and no significant difference was observed between the two regions [40, 41].

Patients with T2DM demonstrate lower RBF and higher ectopic fat deposition than healthy subjects, despite comparable eGFR. These findings suggest that both hemodynamic impairment and renal ectopic fat accumulation are early and persistent contributors to the natural history of DKD, with the latter playing a crucial role in disease progression. The combined use of ASL (for hemodynamic assessment) and IDEAL-IQ (for quantifying ectopic fat) represents a promising approach to detecting early alterations and monitoring disease progression.

In addition, this study provides further evidence for the ability of ASL and IDEAL-IQ to detect changes in renal perfusion and ectopic fat deposition across the full spectrum of renal disease, from early to end-stage. Significant differences in renal cortical and medullary perfusion, as well as fat deposition, were observed between HCs and subgroups of patients with diabetes. As kidney failure progressed, renal perfusion decreased while ectopic fat increased in patients stratified by DKD stage [42]. These findings have important implications for the early diagnosis and staging of DKD, suggesting that RBF and PDFF could serve as potential biomarkers for detecting incipient and subclinical kidney damage, as well as predictors of DKD progression. The noninvasive nature of the quantitative MRI techniques used in this study, combined with their ability to provide comprehensive physiological information, highlights their value for diagnosing, prognosticating, guiding drug development, and monitoring therapy in DKD within a clinical setting.

Accurate evaluation of the hemodynamic effects of ectopic fat deposition on the renal vascular system is critical for clinical management. In this study, a significant negative correlation was observed between RBF and the accumulation of fat in the renal parenchyma and perirenal area. Renal ectopic fat deposits are associated with renal vasculature damage, glomerular sclerosis, tubular damage, interstitial fibrosis, and ultimately impaired renal function [7, 14, 43, 44]. In addition, an experimental study on renal ectopic fat deposition and renal hemodynamics showed that renal ectopic fat deposition contributes to renal perfusion damage, primarily through mechanical compression and synthesis of adipokines [15]. We also found a positive correlation between cortical RBF values and eGFR in T2DM patients. Overall, an increase in renal parenchymal fat deposition may play an important role in decreased renal perfusion observed in patients with DKD. The early detection of these changes could guide the development of novel therapeutic targets to improve renal perfusion and prevent the onset of renal functional impairment.

This study was subjected to certain technical constraints. The measurement protocol was restricted to a single anatomical slice, which may lead to an underestimation of both overall renal perfusion and ectopic fat deposition. Moreover, while this investigation employed a cross-sectional design, future longitudinal studies with larger cohorts are needed to evaluate the clinical utility of these markers for prognostic stratification and therapeutic interventions.

## Conclusion

In conclusion, our study demonstrated the clinical utility of quantitative MRI techniques, including ASL for renal perfusion evaluation and IDEAL-IQ for ectopic fat deposition measurement, in assessing renal functional impairment and disease progression in patients with T2DM. We identified a significant tripartite correlation between reduced renal perfusion, elevated ectopic fat accumulation, and DKD progression, indicating that these factors synergistically drive renal functional decline. In addition, ectopic fat deposition was independently associated with renal hemodynamic injury. These findings highlight the potential of quantitative MRI as a noninvasive multimodal tool for early DKD diagnosis, risk stratification, and therapeutic targets identification aimed at restoring renal perfusion and mitigating fat-driven parenchymal damage.

## Data Availability

The datasets used or analyzed during the current study are available from the corresponding author upon reasonable request.

## Abbreviations

ASL: Arterial spin labeling
AUC: Area under the curve
BMI: Body mass index
DKD: Diabetic kidney disease
DM: Diabetes mellitus
eGFR: Estimated glomerular filtration rate
FKD: Fatty kidney disease
FOV: Field of view
HC: Healthy control
ICC: Intraclass correlation coefficient
IDEAL-IQ: Iterative decomposition of water and fat with echo asymmetry and least-squares estimation-iron quantification sequence
MRI: Magnetic resonance imaging
PDFF: Proton density fat fraction
RBF: Renal blood flow
ROC: Receiver-operating-characteristic
ROIs: Region of interest
Scr: Serum creatinine
T2DM: Type 2 diabetes mellitus
TE: Echo time
TR: Repetition time
UACR: Urine albumin creatinine ratio
